# Determinant of unmet need for family planning among adolescent and young women in Kenya: multilevel analysis using recent Kenyan demographic health survey

**DOI:** 10.3389/frph.2025.1511606

**Published:** 2025-02-04

**Authors:** Beyene Sisay Damtew, Hinsermu Bayu Abdi, Beker Ahemed Hussien, Getahun Tiruye, Nafyad Tolossa Urgie, Beniam worku Yigezu, Sifan Ahmed Mohammed, Bezawit Melak Fente

**Affiliations:** ^1^ Department of Midwifery, College of Health Science, Arsi University, Asella, Ethiopia; ^2^ Department of Biomedical Science, College of Health Science, Arsi University, Asella, Ethiopia; ^3^ Department of General Midwifery, College of Medicine and Health Science, Gondar University, Gondar, Ethiopia

**Keywords:** unmet need, family planning, adolescent, young age, Kenya

## Abstract

**Background:**

Unmet need for family planning (FP) refers to the proportion of women who are fecund, sexually active, and wish to delay or limit childbearing but are not using any effective contraceptive method. Unmet need for FP remains a significant public health concern, particularly among young women aged 15–24 years. This study explores the determinants of unmet need for FP among young women.

**Method and material:**

This study used data from the 2022 Kenyan Demography and Health Survey to investigate the relationship between various factors and a binary outcome variable of unmet need for family planning among adolescent young age group. A multilevel binary logistic regression model was used to analyze the data, accounting for the clustering effects of the survey data. The statistical significance of the relationships was assessed using adjusted odds ratios with 95% confidence intervals. The model with the lowest deviance was considered the best fit for the data.

**Result:**

Magnitude of unmet need family planning among adolescent young age in Kenya is 25% (95% CI: 24.5%–26%). Determinants of Unmet need for family planning are age 15–19 (AOR: 3.4, 95%CI (1.3–11), undecided desire number of children (AOR: 2.3, 95%CI: 1.4–2.1), age at first sex 15–29 (AOR: 2.7, 95%CI: 1.2–6.2), rural residency (AOR: 3.9, 95%CI: 1.1–14), high community level poverty (AOR: 2.1, 95%CI: 1.1–4.5).

**Conclusion:**

The study finds that 25% of young women in Kenya lack access to family planning. Factors like age, desired family size, early sex, rural life, and poverty contribute to this. These factors create barriers that hinder women's ability to plan their pregnancies and access necessary resources. Younger women, those in rural areas, and those living in poverty are particularly affected. Addressing this issue requires a comprehensive approach that considers the specific circumstances of these vulnerable populations.

## Background

Unintended pregnancy remains a significant public health concern globally. Despite advancements in family planning (FP) methods, a substantial unmet need persists, particularly among young women. This unmet need refers to the desire to delay or space pregnancies but not using any effective contraceptive method (1). Understanding the factors that contribute to this unmet need is crucial for developing effective interventions to improve reproductive health outcomes for young women.

Unmet need for family planning (FP) refers to the proportion of women who are fecund, sexually active, and wish to delay or limit childbearing but are not using any effective contraceptive method (2). High levels of unmet need are associated with unintended pregnancies, increased maternal and child mortality, and can hinder economic development (3). Understanding the factors contributing to unmet need for FP is crucial for designing effective interventions to improve reproductive health outcomes, particularly among young women.

Young women in Kenya face a unique set of challenges in accessing and utilizing FP services. Despite significant progress in reducing fertility rates nationally (4), unmet need for FP remains a significant public health concern, particularly among young women aged 15–24 years (5). This age group is particularly vulnerable due to factors such as limited knowledge about contraceptive options, sociocultural barriers, economic constraints, and power imbalances within relationships (6).

This study aims to identify the determinants of unmet need for FP among young women in Kenya. We utilize data from the most recent Kenyan Demographic and Health Survey (KDHS) to explore the socio-demographic, economic, and behavioral factors associated with unmet need (7, 8). By analyzing these factors, this study seeks to contribute to a deeper understanding of the challenges young women face in achieving their reproductive desires and inform the development of targeted FP interventions that address their specific needs.

The majority of teenage girls and young women in sub-Saharan Africa are growing up in underprivileged environments characterized by high rates of unemployment, fast urbanization, few educational options, and quickly shifting sociocultural norms and procedures (9). In addition to these general difficulties, they deal with a variety of sexual and reproductive health problems, including high fertility, unsafe abortions, and unwanted pregnancies (9, 10). Family planning (FP), is a way to empower women, improve health, and fight poverty (11, 12). By enabling women to postpone motherhood, spacing out pregnancies, avoid unplanned pregnancies and abortions, and cease having children when they have reached their ideal family size, it can avert up to one in three maternal deaths (12, 13). 25% of married women in SSA who are of reproductive age have unmet fertility need (14). A significant proportion of adolescent young women in developing nations like Kenya lack access to adequate family planning services (15). In contrast to other research, the current study employed multilevel analysis to model the hierarchical nature of the data.

Different study done in Ethiopia, Burundi shows that rural residency is among main factors determined for unmet need for family planning (16–18). Study from systematic review done in Ethiopia shows age at 1st marriage less than 18, women with no formal education, no discussion with partner is a predictors for unmet need for family planning (13). Study from scope review shows that age and level of education was negatively associated with unmet need for family planning while number of child positively associated with unmet need for family planning (19).

The unmet need for family planning arises from various factors, including limited access to healthcare facilities, inadequate knowledge about contraceptive methods, cultural and religious barriers, health concerns, and partner disapproval (19, 20). This unmet need leads to unintended pregnancies, which can have severe consequences such as increased maternal and child mortality, poverty, and limited opportunities for women (21). Addressing these factors through improved access to family planning services, education, and counseling is crucial for promoting women's health, reducing poverty, and achieving sustainable development.

This study explores the determinants of unmet need for FP among young women. It examines various socio-demographic, economic, and knowledge-based factors that may influence young women's access to and utilization of FP services. By identifying these determinants, this study aims to inform the development of targeted strategies to address the unmet need for FP and empower young women to make informed choices about their reproductive health.

## Method and material

### Data source

We utilized data from the 2022 Kenya Demographic and Health Survey (KDHS), which was the seventh survey of its kind conducted in Kenya. This data was obtained through a formal request to the Measure DHS program (https://dhsprogram.com/Data/terms-of-use.cfm). The KDHS employed a two-step sampling design, selecting 12,166 clusters from the Kenya Household Health Survey Framework using a random selection method. We focused on the individual records dataset (IR file), which contains information on unmet need family planning women of adolescent young age.

### Study population

The study population focused on Adolescent and young women aged 15–24 years who are currently married or in a union. We excluded women who are pregnant at the time of the survey or are sterilized.

Exclusion: In-fecund, pregnant, and women age 25–49.

### Outcome Variable

The primary outcome variable was unmet need for family planning. In the DHS the question of unmet need for family planning was answered by “are you a women who are fecund, not pregnant, and want to delay or space your next childbirth but unable to access any effective contraceptive method?” all respondent's response of “yes” are considered as unmet need for family planning and coded as “1” those respondent's response of “no” are met need for family planning and coded as “0” (22).

### Explanatory variables

Explanatory factors considered included a woman's age (15–19, 20–24), educational level (no formal education, primary school, secondary), working status (not working, working), and number of children (0, 1–2, 3–4, 5+). Media exposure (newspaper, radio, or TV) was assessed based on frequency (no, yes), wealth level (poorest, poorer middle, richer, richest), distance to a health facility (big problem, not a big problem), place of residence (urban, rural), community literacy level (low, high), community poverty level (low, high), and community media exposure level (low, high).

### Statistical analysis

Descriptive statistics, including frequency and percentage distributions, were used to characterize respondents and their unmet family planning needs. Bivariate multilevel logistic regression analyses were then conducted to identify variables significantly associated with unmet needs at a *p*-value of less than 0.25. A multi-collinearity test, using the variance inflation factor (VIF), was performed on all statistically significant variables from the bivariate analyses. To evaluate the impact of individual/household and community-level factors on unmet family planning needs, four multilevel logistic regression (MLLR) models were constructed (23). Model 0: A null model with no explanatory variables, demonstrating the baseline variance in unmet needs. Model I: Included individual/household-level factors. Model II: Included community-level factors. Model III: A complete model incorporating both individual/household and community-level factors.

A two-level multilevel binary logistic regression model is a statistical technique used to analyze hierarchical data with two levels individual with communities.

$$\mathrm{logit}(\pi_{\mathrm{ij}}=1)=\beta_{0}+\beta_{1}X_{1}+\ldots +\beta_{k}X_{\mathrm{kij}}+u_{0j}+\varepsilon_{\mathrm{ij}}$$

Where, π_ij_: The probability of unmet need for family planning, *β*₀: The overall intercept. *β*₁ to *β*_k_: Regression coefficients indicating a unit increase in X cause a ß unit increase probability unmet need for FP. X₁_ij_ to X_kij_: individual level predictors k for individual i^th^ in community j^th^. u₀_j_: shows the random effect for the j^th^ cluster, *ε*_ij_: The individual-level error term (22, 24, 25).

## Results

### Socio-demographic related factors

A majority of the adolescent young women (52.6%) are aged 15–19. A significant proportion (62.4%) of the young women resides in rural areas. Majorities (64.5%) of Adolescent young women have secondary education and above educational level. Wealth distribution among the adolescent young women is relatively even, with the poorest category accounting for 23.1% of the sample, the poorer category accounting for 19.3%, the middle category accounting for 19.3%, the rich category accounting for 21.8%, and the richest category accounting for 15.8% (Table 1).

**Table 1:** Socio-demographic factors of unmet need for family planning among adolescent young women in Kenya, KDHS 2022.

Variables	Frequency	Unmet need family planning	
		Yes	No
Age			
15–19	6,404 (52.6)	656	5,748
20–24	5,762 (47.4)	2,427	3,335
Education level			
No education	789 (6.5)	65	724
Primary	3,524 (29)	836	2,688
Secondary and above	7,853 (6.5)	2,182	5,671
Maternal working status			
Not working	3,402 (27.9)	1,452	1,950
Working	8,753 (72.1)	1,625	7,128
Wealth status			
Poorest	2,812 (23.1)	540	2,272
Poorer	2,351 (19.3)	562	1,789
Middle	2,439 (19.3)	612	1,827
Rich	2,647 (21.8)	811	1,836
Richest	1,917 (15.8)	558	1,359
Distance from health facility			
No big problem	4,806 (75.1)	1,252	3,554
Big problem	1,595 (24.9)	350	1,245
Media exposure			
Yes	3,745 (30.8)	917	2,828
No	8,421 (69.2)	2,166	6,255
Residence			
Urban	4,579 (37.6)	1,295	3,284
Rural	7,587 (62.4)	1,788	5,799
Community media			
High	331 (50.1)	56	274
Low	330 (49.9)	54	277
Community education			
High	166 (74.1)	82	413
Low	495 (25.9)	28	138
Community poverty			
High	330 (49.9)	64	266
Low	331 (50.1)	46	285

### Reproductive and contraceptive related factors

A significant portion of women (59.4%) had their first sexual encounter at age 15–19. Nearly half (47.3%) gave birth for the first time at age 15–19. Most women (65%) haven't had any children yet, while 31.7% have 1–2 children. Only a small percentage (3.9%) has 3 or more children. The desire for children is high, with 81% of women wanting them. However, 10.9% are undecided, and 8.1% don't want children. A large majority (74.9%) have heard about family planning methods, suggesting potential openness to contraception. Caesarean sections (C-sections) are uncommon, with only 11.1% of deliveries happening this way (Table 2).

**Table 2:** Reproductive and contraceptive related factors for unmet need family planning among adolescent young women in Kenya, KDHS 2022.

Variables	Frequency	Unmet need family planning	
		Yes	No
Age at first sex			
15–19	7,221 (59.4)	845	6,376
20–24	945 (40.6)	2,238	2,707
Age at first birth			
15–19	2,044 (47.3)	1,094	1,187
19–24	2,281 (52.7)	1,082	962
Total number of child			
No child	7,910 (65)	921	6,989
1–2	3,857 (31.7)	2,000	1,857
3–4	374 (3.1)	156	222
5+	21 (0.8)	6	15
Desire for children			
Yes	5,185 (81)	206	311
Undecided	699 (10.9)	68	631
No	517 (8.1)	1,328	3,857
Delivery by C/S			
Yes	385 (11.1)	215	170
No	3,070 (88.9)	1,519	1,551
Heard about family planning			
Yes	4,795 (74.9)	1,431	3,364
No	1,607 (25.1)	171	1,436

### Prevalence of unmet need for family planning among adolescent and young women in Kenya

**Figure 1:**
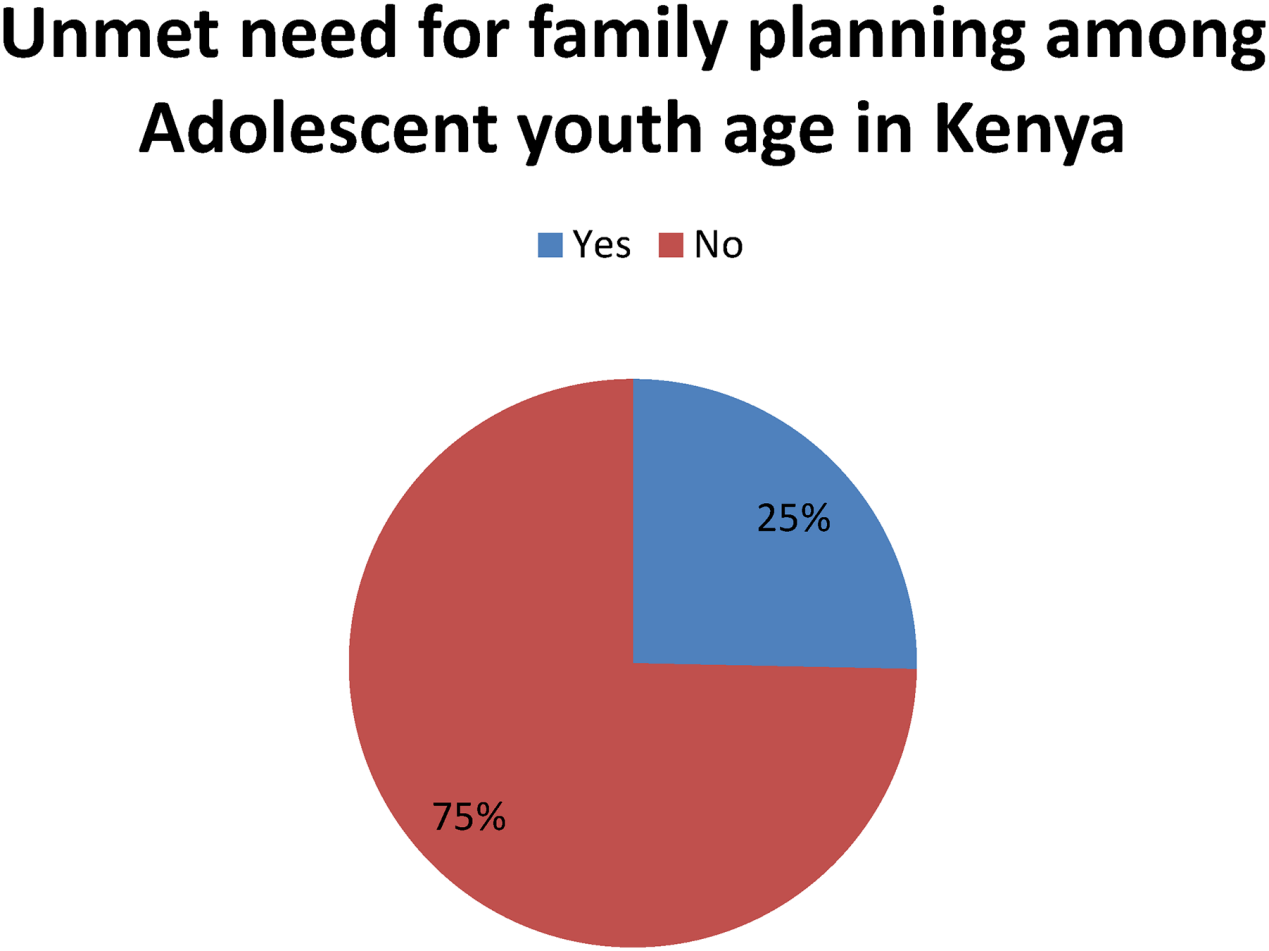
Magnitude of unmet need for family planning among adolescent young women in Kenya, KDHS 2022.

The finding shows that 25%25% (95% CI: 24.5%-26%) of adolescent youth in Kenya have an unmet need for family planning, while 75% meet their need for family planning. This indicates that significant portions of adolescent youth in Kenya are not receiving the necessary family planning services to meet their reproductive health needs (Figure 1).

### Determinants of unmet need for family planning among adolescent young women in Kenya

In the multivariable mixed effect binary logistic regression model, age of respondent, desire for children, and age of first birth significant individual factors while place of residence, and community-level education were found to be statistically significant factors from community-level factors of unmet need for family planning among Kenya young and adolescent age**.**

Adolescent and young women aged 15–19 were significantly more likely to have unmet family planning needs than those aged 20–24 (AOR: 3.4, 95% CI: 1.3–11). Women who were undecided about wanting children were also more likely to have unmet needs compared to those who did (AOR: 2.3, 95% CI: 1.4–2.1).

Furthermore, adolescents who had their first sexual experience between the ages of 15 and 19 were 2.7 times more likely to have unmet family planning needs than those who waited until age 19–24 (AOR: 2.7, 95% CI: 1.2–6.2). Women living in rural areas were 3.9 times more likely to have unmet family planning needs compared to those living in urban areas (AOR: 3.9, 95% CI: 1.1–14). As well as women living in communities with high levels of poverty were 2.1 times more likely to have unmet needs than those in low-poverty communities (AOR: 2.1, 95% CI: 1.1–4.5) (Table 3).

**Table 3:** Determinant of unmet need for family planning among adolescent young women in Kenya, KDHS 2022.

Variables	Null model	Model I	Model II	Model III
Age of respondent				
15–19		0.6 (0.49–0.85)		**3.4** (1.3–11)*
20–24		1		1
Occupation				
Working		2.3 (1.8–2.9)		0.7 (0.34–1.8)
Not working		1		1
Media exposure				
Has exposure		0.9 (0.7–1.2)		0.7 (0.2–2.2)
No exposure		1		1
Total number of child				
No child		1		1
1–2		0.1 (0.07–0.17)		1.2 (1–4)
3–4		0.2 (0.08–0.26)		0.4 (0.1–1.2)
5+		0.08 (0.07–0.4)		0.2 (0.21–1.1)
Distance from health facility				
Not a big problem				1.1 (0.5–2.5)
A big problem		0.6 (0.4–0.8)		1
Desire for children				
Yes		1		1
Undecided		2 (1.2–3.5)		**2.3** (1.4–2.1)*
No		0.8 (0.6–1.3)		1.2 (0.3–3.3)
Heard about family planning				
Yes		1		1
No		2 (1.4–2.8)		1.2 (0.5–3.4)
Age at first sex				
15–19		0.1 (0.48–0.84)		0.2 (0.09–1.2)
20–24		1		1
Age at first birth				
15–19		0.13 (0.68–1.2)		**2.7** (1.2–6.2)**
20–24		1		1
Husband's desire for children				
Both want same		1		1
Husband wants more		1.2 (0.87–1.5)		2 (0.6–2.2)
Husband wants fewer		0.7 (0.45–1.2)		0.3 (0.5–3.3)
Don't know		1.4 (1.1–2.1)		2.1 (0.5–1.6)
Residence				
Rural			0.6 (0.39–0.94)	**3.9** (1.1–14)*
Urban			1	1
Community education				
Low			1.2 (0.7–1.9)	1.6 (0.6–4.1)
High			1	1
Community level of poverty				
Low			1	1
High			1.4 (0.9–2.2)	**2.1** (1.1–4.5)*
Community media exposure				
Low			1	1
High			1.05 (0.7–1.6)	0.7 (0.3–1.5)

Bold indicate the variable is significant.

* Indicate *p*-value is ≤0.05.

** Indicate *p*-value is <0.001.

### Random effect result for unmet need among adolescent young age women of Kenya

The statistical analysis revealed that the best-fitting model for predicting the likelihood of unmet need for family planning was the one with the lowest deviance value (110.41106) and AIC (142.4111). The inclusion of individual/household and community level factors in the model significantly improved its fit. The ICC values showed that a substantial portion of the variation in unmet need for family planning can be attributed to differences between primary sampling units (clusters). This suggests that factors at the community level play a significant role in determining the likelihood of unmet need for family planning. As more factors were included in the model, the between-cluster variation increased, indicating that the clustering effect becomes more pronounced with the inclusion of additional predictors (Table 4).

**Table 4:** Random effect result for unmet need among adolescent young age women of Kenya, KDHS 2022.

Random effect	Null model	M1	M2	M3
Log-likelihood	−6,720.8255	−1,033.5396	−293.05439	−55.205,532
ICC (%)	12.9	17.3	23	31.2
AIC	13,445.65	2,107.079	596.1088	142.4111
BIC	13,460.46	2,216.767	618.5775	183.6064
Deviance	13,441.651	2,067.0792	586.10878	110.41106
PCV	Ref	19	54	58
Wald x^2^ and *p*-value	Ref	X^2^ = 225.87, *P* < 0.001	X^2^ = 8.74, *p* < 0.001	X^2^ = 9.94, *p* < 0.001

## Discussion

This study assessed the magnitude and determinant of unmet need for family planning among adolescent and young age group of Kenya women. The current study shows that 25% (95% CI: 24.5%–26%) of them has unmet need for family planning. Almost one third of adolescent young women in Kenya has unmet need for family planning. This study shows lower than study conducted in Ethiopia 28% and Tanzania 31.8% (22, 26). This lower prevalence may be due to that in Kenya, a more equitable distribution of healthcare facilities, particularly in rural areas, coupled with positive cultural attitudes towards family planning, might make it easier for young women to access and utilize contraceptive services. Additionally, robust government programs and policies aimed at promoting family planning and improving access to contraception could play a significant role. However the study shows high unmet need than study conducted in Guinea 22.6% and India 9.4% (27, 28). Even though Kenya may have strong government policies and programs supporting family planning, implementation challenges or limited resources could still impact access to services.

Age 15–19 is significantly associated with unmet need for family planning among adolescent and young age (AOR: 3.4, 95%CI; 1.3–11). This is consistent with study conducted in Sierra Leone and Guinea (29, 30). This consistency may due to early marriage and childbearing are common in many regions which increase the risk of unintended pregnancies. Additionally, adolescents are more vulnerable to sexual exploitation and coercion, which can lead to unwanted pregnancies and limited access to family planning services.

Those mother undecided number of children has significantly associated with unmet need for family planning in Kenya (AOR: 2.3, 95%CI: 1.4–2.1). Consistent study are found in Congo and multilevel analysis in East Africa shows the as the number of family increase and undecided number of children significantly associate with unmet need for family planning (7, 8, 31, 32). Those studies have found that as the desired number of children increases, so does the likelihood of experiencing unmet need for family planning. This suggests that women who are unsure about how many children they want may be less likely to take proactive steps to plan their pregnancies, leading to unintended pregnancies and unmet need.

Adolescent young women who experience sex at age 15–19 significantly associate with unmet need for family planning (AOR: 2.7, 95%CI: 1.2–6.2). This study supported by study done in Washington (33). This might be because younger people (15–19 years old) are more likely to not have access to modern contraception, which could lead to more unintended pregnancies among those who are sexually active.

The study revealed that adolescent young age women live in rural are significantly associated with unmet need for family planning (AOR: 3.9, 95%CI: 1.1–14). This finding is consistent with study done in different part of Ethiopia, Burundi (16, 17, 34, 35). This consistent finding may be due to significant socio-cultural barriers, limited access to healthcare, and lack of education faced by adolescent young women in rural areas, which contribute to their unmet need for family planning. In addition, early marriage, traditional gender roles, and limited knowledge about family planning methods in rural area may restrict women's autonomy and decision-making power. Moreover, Geographic isolation, a shortage of trained healthcare providers, and financial constraints, coupled with lower education levels and limited economic opportunities, contribute to the increased vulnerability of these women to early marriage and unplanned pregnancies which may contribute to unmet need for family planning. There is contrary finding reported from Ghana (36). This may be due to the high level of awareness about family planning services in rural Ghana which has, likely contributed positively to their uptake, despite potential challenges in accessing these services.

High community level poverty is significantly associated with unmet need for family planning among adolescent and young women (AOR: 2.1, 95%CI: 1.1–4.5). Consistent studies were reported from Ethiopia, Congo, Uganda, Pakistan (11, 31, 34, 37, 38). This consistency may due to the fact that poverty and limited income can significantly hinder women's access to and affordability of family planning services. Financial constraints can prevent women from paying for contraceptives, traveling to healthcare facilities, or even consulting with healthcare providers. In addition, women living in poverty may prioritize other essential needs, such as food, shelter, and clothing, over family planning services. Furthermore, poverty can be associated with limited access to information about family planning methods and their benefits, as well as a lack of trust in healthcare providers.

### Strengths and limitations

The study offers a comprehensive examination of the factors influencing unmet need for family planning among adolescent and young women in Kenya. It is based on a nationally representative sample, ensuring the generalizability of its findings. Additionally, the study compares Kenya's situation to other countries, providing valuable insights into its performance in this area.

As a cross-sectional study, this research provides a snapshot of the situation at a specific point in time, limiting its ability to establish causality or track changes over time. Moreover, the reliance on self-reported data introduces the potential for bias. Finally, while the study identifies government policies and programs supporting family planning, it provides limited detail on the challenges faced in implementing these initiatives.

## Conclusion

The study shows 25% unmet need for family planning among adolescent young women in Kenya. Factors such as their age, desired number of children, age of sexual activity, place of residence, and socioeconomic status are significantly associated. These factors intersect to create a complex web of challenges that limit women's ability to plan their pregnancies and access necessary resources.

Specifically, younger women, those in rural areas, and those living in poverty are disproportionately affected by unmet need for family planning. This is likely due to a combination of limited access to healthcare, cultural barriers, and economic constraints. Addressing this issue requires a comprehensive approach that takes into account the unique circumstances of these vulnerable populations.

### Implication of the study

The study emphasizes the urgent need for targeted interventions to address the unmet need for family planning among adolescent and young women in Kenya. Prioritizing this population requires expanding access to healthcare services, strengthening government programs, challenging harmful cultural norms, reducing poverty, and conducting further research to inform effective interventions. By implementing these measures, Kenya can significantly improve the reproductive health and well-being of its adolescent and young women.

## Data Availability

The original contributions presented in the study are included in the article/Supplementary Material, further inquiries can be directed to the corresponding author.
